# Qualitative exploration of determinants of active mobility and social participation in Urban neighborhoods: individual perceptions over objective factors?

**DOI:** 10.1186/s13690-024-01408-z

**Published:** 2024-10-16

**Authors:** Lukas Bollenbach, Martina Kanning, Christina Niermann

**Affiliations:** 1 Department of Social and Health Sciences in Sport Science, University of Konstanz, Konstanz, Germany; 2 Institute of Interdisciplinary Exercise Science and Sports Medicine, Medical School Hamburg, Hamburg, Germany

**Keywords:** Active transport, Physical activity, Exercise, Social environment, City, Walkability, Qualitative study, Urban health

## Abstract

**Background:**

Urban neighborhood environments play an important role in facilitating or hindering residents to engage in active mobility and social participation. However, while there is much quantitative research, in-depth knowledge that contextualizes residents’ subjective perceptions of barriers and facilitators of active mobility and social participation is still insufficient. Therefore, a qualitative approach was used to collect subjectively perceived barriers and facilitators of active mobility and social participation of residents from different neighborhoods with objectively determined high vs. low walkability. Furthermore, to better understand (non) concordance of objective environmental characterizations and actual levels of behavior, low and high walkability neighborhood-specific barriers, proposed improvements, and particularities that determine (non) engagement in active mobility and social participation were explored.

**Methods:**

Three focus groups (*N* = 6, *N* = 6, and *N* = 5) with 17 participants (7 women, 10 men) aged 21–64 (mean age 43.4 ± 14,6 years) were conducted utilizing a pre-structured interview guideline. Participants lived in 11 different neighborhoods with either high or low objectively determined walkability. The focus groups were transcribed verbatim, followed by a thematic analysis of the content with deductive and inductive code categories, utilizing the MAXQDA software.

**Results:**

Notable was the consensus of many perceived barriers and facilitators of active mobility and social participation along with their assignability to the same context (points-of-interest, infrastructure; safety, communication, community; topography, physical compositions, weather, aesthetics; personal / individual attitudes, influences, evaluations). Another main finding was that high and low walkability neighborhood-specific particularities were revealed that are in contrast to some objective characterizations of walkability: For example, too high density can inhibit active mobility, and too many options can inhibit social participation.

**Conclusions:**

The consensus of many barriers and facilitators of active mobility and social participation suggests that valuable synergies could be created by coordinating interventions aiming to promote both active mobility and social participation in urban neighborhoods. Also, considering subjective perceptions of residents helps to identify neighborhood-specific factors that determine (non) engagement in active mobility and social participation. The findings can help city planners and public health officials improve the promotion of active mobility and social participation in the creation of health-enhancing urban neighborhoods.

**Supplementary Information:**

The online version contains supplementary material available at 10.1186/s13690-024-01408-z.

**Table:** 

Text box 1. Contributions to the literature
• Active mobility and social participation play an important role in the context of health-promoting urban neighborhoods, as they can positively influence the physical, mental, and social health of residents.
• Implementing qualitative focus groups allows to gather in-depth insights about barriers and facilitators of active mobility and social participation in urban neighborhoods.
• Considering subjectively perceived barriers and facilitators of active mobility and social participation can help to understand (non) concordance between objective environmental characterizations and actual levels of health behavior.
• High and low walkability neighborhood-specific investigations can offer valuable insights that speak against one-size-fits-all approaches in promoting active mobility and social participation.

## Background

In the EU, insufficient levels of physical activity across all ages of the population have a considerably negative effect on population health [1]. The consequences of insufficient levels of physical activity are manifold and detrimental to health, making its promotion important. In this context, active mobility (AM, physical activity that’s undertaken to travel from A to B to reach a destination, e.g., walking and biking for leisure, recreation, errands, transport, etc. [2]) can increase individuals’ overall physical activity levels and thereby help to reach the WHO’s recommendation for health-promoting levels of physical activity [3, 4]. In addition, AM is, at least for physically not restricted individuals, a highly accessible way to get from A to B, making its promotion valuable for a wide range of the population, especially in cities [5–7]. Moreover, AM is also associated with everyday social participation (SocPar, being involved in activities that result in interaction with other individuals [8]) by increasing accessibility and chances for social interactions [9–11]. This is of great relevance, as more and more individuals who live in cities experience loneliness and social isolation, which is detrimental to health [12]. With this in mind, not only does research indicate that promoting and supporting individuals to increase their levels of AM is associated with increased SocPar, but also vice versa [13–15]. Furthermore, an increase in AM and SocPar can benefit urban health through more social encounters and -interactions, greater physical activity levels, higher well-being, as well as less traffic, air pollution, noise, and temperature related to motorized traffic, and many more [5, 16–20]. It follows that fostering both AM and SocPar is a promising and valuable health-promoting strategy for urban environments.

However, to promote AM and SocPar, it’s important to understand their determinants and correlates. In this context, social-ecological models posit that individuals’ (non) engagement in a behavior, for example, AM / SocPar, is a result of determinants from different dimensions that mutually influence each other: E.g., the environment, the individual, and the interaction between individuals and their environment [21–23]. This is supported by empirical evidence that has shown that individuals’ AM and SocPar in urban environments depend on various factors: On the one hand, the built- (e.g., availability of amenities and infrastructure [24, 25]), natural- (e.g., greenspaces and parks [26, 27]), and social (e.g., population density, social interactions [28, 29]) environment are relevant [30–32]. On the other hand, the individuals themselves (e.g., their attitudes, subjective perceptions, resources, etc. [29, 33]), and how they interact with different environments, have to be considered as well [34, 35]. One important study in this context is the systematic review by Salvo et al. [28] that included 36 peer-reviewed qualitative studies. The review summarized the influence of the built environment (functional, aesthetic, destination, and safety characteristics), but also social environment (e.g., social interaction, sense of community) on the decision to engage in walking, biking, strolling, active transportation, and more. The findings underline the necessity and value of considering different (environmental) factors in the creation of PA promoting neighborhoods and support the need to include residents in this process. Another important study in this regard is by Strobl et al. [25], who conducted 11 focus groups with 78 individuals to investigate the relevance of the structure of a community and the characteristics of a neighborhood for SocPar. They asked participants to detail their community activities and to identify barriers and facilitators to SocPar. Important findings were the importance of a well-designed infrastructure, and neighborhood social cohesion and community for SocPar. However, despite different theoretical and empirical approaches and investigations on determinants of (non) engagement in AM and SocPar, actual rates of individuals engaging in AM and SocPar remain low: In the EU, only one in three adults comply with the WHO’s physical activity recommendations, and more than a third of participants from an EU-wide study reported to be lonely at least sometimes (13% reported to be lonely most of the time) [1, 36]. One reason for this is that often, interventions intended to promote health behavior solely focus on pre-determined, objective factors, and neglect the concurrent importance of individuals’ perceptions and evaluations [37, 38]. Therefore, in aiming to promote AM and SocPar, it’s necessary to gain more knowledge about how residents perceive and evaluate facilitators and barriers of AM and SocPar in their respective neighborhood environments.

To do so, it’s useful to use a consistent characterization of the neighborhood environments, upon which residents’ perceived facilitators and barriers can then be investigated. In this context, a prominent construct that typically describes aspects of the environment related to AM and SocPar objectively, but also allows for subjective assessment, is walkability [39]. Walkability can describe the availability of amenities, pedestrian network, proportion of greenspaces, population density, slope, and more in a given area to determine how friendly that area is for AM and SocPar [40, 41]. Specifically, high walkability can indicate a good-, and low walkability can indicate bad accessibility, friendliness, and possibilities for individuals to engage in AM and SocPar. Generally, associations between walkability and AM are largely supported by empirical findings (e.g., [42]). Yet, there are also inconsistencies between objective and subjective walkability assessments and their association with AM: For example, the study from Arvidsson et al. [43] shows broad concordance between both objective and subjective assessments of walkability and actual engagement in AM. Contrarily, the study by Gebel, Baumann, and Owen [44] reports discordance. Similarly, concerning SocPar, empirical findings generally support associations between walkability and SocPar (e.g [40]). But, also in this context, inconsistencies can be found: Jun and Hur [45] reported findings that suggest a positive association between perceived walkability and SocPar, but a negative association between objective walkability and SocPar. These inconsistencies in the findings can be attributed to subjective perceptions capturing different aspects of the environment than objective determinations [46, 47]. This is in line with other research that indicates that objective measures of urban characteristics often don’t match individuals’ subjective perceptions [48].

A possible explanation is that individuals’ engagement in AM and SocPar depends on what they perceive and evaluate to be facilitating and hindering factors. However, what a facilitator or barrier for AM or SocPar is, or how it’s perceived, may not always result in consistent behavior across different settings (e.g. high vs. low walkability), different individuals, or different situations. Therefore, to better understand residents’ (non) engagement in AM / SocPar, more knowledge is needed about what they perceive as facilitators or barriers. Moreover, insights about possible differences in that perception due to different neighborhood conditions or circumstances, for example, high vs. low walkability neighborhood environment, are necessary.

In light of this, qualitative explorations can offer valuable in-depth insights into health behaviors along with interactions between individuals and the respective neighborhood environment [49]. Furthermore, they allow to address individual evaluations like improvements for barriers and facilitators [50]. Putting this to use, this study conducted qualitative focus groups with adults from different urban neighborhoods with objectively determined high or low walkability. This was done to investigate and compare neighborhood-wide (non) concordance of factors for (non) engagement in AM and SocPar. Furthermore, neighborhood-specific barriers for AM and SocPar engagement, along with suggested improvements for barriers and facilitators, and particularities were explored. The results of this study can aid city planners and public health officials to better understand, why and under which circumstances urban residents do or do not engage in AM and SocPar in the context of health-promoting urban environments.

## Objectives

This study has the following objectives:

1. To collect general key factors (barriers, facilitators) that residents state to determine their (non) engagement in AM and SocPar.
2. To investigate high and low walkability neighborhood-specific barriers and peculiarities along with an exploration of suggestions for improvements of urban dwellers.

### Methods

#### Study area and participants

The three focus group interviews are part of the research project ‘AMbit - Active Mobility’ (ambit.uni-konstanz.de/) and were conducted in June 2021. Participants were recruited as follows: First, 3000 letters with information about the project AMbit and an invite to participate in an online questionnaire were distributed in 12 neighborhoods of the city of Stuttgart, Germany. The distribution was carried out by project members who physically delivered the letters while walking through the streets of the respective neighborhoods. The neighborhoods were pre-selected to ensure an even allocation of participants into six high and six low walkability neighborhoods. The following neighborhoods were chosen: Birkach, Degerloch/Haigst, Feuerbach-Ost, Feuerbach-West, Möhringen, Mönchhalde, Kaltental, Kräherwald, Ostheim, Plieningen, Untere Birkenwaldstraße, and Vaihingen. The classification of the neighborhoods’ walkability into high and low was derived from the first version of the ‘Walkability-Index’ of the ‘Research Institute for Regional and Urban Development’ (ILS) [41]. The index used the variables permeability of the pedestrian network, proportion of green spaces, population density, and availability of amenities within walking distance to determine the walkability. For a comprehensive and detailed description of the neighborhood classification process, including variables, scaling, calculation, and high / low walkability categorization, please see the method section in the publication from Bollenbach et al. [48]. In a second step, those who completed the questionnaire had the chance to opt in to be contacted via email to participate in the focus group interview study. Optional 20 € were offered as an incentive to participate in the focus group, provided participants chose to share their bank details with the project team for the sole purpose of transferring the money. The bank information was deleted immediately after the incentive was transferred. Stuttgart has the particular feature of being located in a valley basin, which results in the neighborhoods being located in a variety of topographies, for example, hillside locations with slopes, flat, urban, more rural, etc. This allowed the inclusion of many different neighborhoods with different characteristics. The final sample consisted of a total of 17 individuals (11 individuals from high-, and 6 individuals from low walkability neighborhoods) from 11 different neighborhoods. Inclusion criteria were to be at least 18 years of age, speak German, and live in one of the residential neighborhoods of Stuttgart. Study participation was voluntary and the subjects were able to withdraw at any time without stating a reason. Participants received written and oral information about the study background, aims, procedure, rights, and data protection before the focus groups. Also, before the start of the focus groups, individuals gave written and oral consent to the participation in and the recording (video / audio) of the focus groups. At the time of the focus groups, the COVID-19 pandemic was still somewhat an issue and to avoid possible uneasiness of meeting face to face, the focus groups were conducted via the online tool Zoom [51]. Using Zoom further facilitated the scheduling of times and dates of the focus groups. The focus groups were conducted in German language.

## Procedure of the focus group interviews

*N* = 3 focus group interviews (G1, *N* = 6; G2, *N* = 6; G3, *N* = 5, participants) were conducted. The participants were allocated to the three focus groups with the goal of an even distribution across the three focus groups in terms of gender and walkability of the neighborhood (high vs. low walkability). However, with regard to participation rates, groups had to be formed based on the best availability of participants on different dates. Nevertheless, a balanced representation of both genders and various walkability areas was achieved. As is common in focus group interviews, a pre-structured interview guideline (see Additional file 1) was used, which enabled a systematic collection and comparison of key factors of AM and SocPar. The interview guide helped to ensure that all questions were asked, that the questions addressed the context of interest, and that the questions were formulated correctly. Furthermore, participants were instructed to keep their own neighborhood in mind when naming barriers and facilitators. Also, this ensured comparability and integration of the answers of the different individuals / focus groups [52]. The interview guideline used in this study was created by the authors of this paper in an iterative process of discussing and testing the questions and implementing feedback loops that included other researchers of the project. Also, a pilot test was run with other researchers from the institute who were not part of the present research project. To ensure a high quality of data collection with the focus groups, the moderator received training regarding the moderation of focus groups. This training included information about possible difficulties that may occur, and how to deal with them, for example, what to do if the focus group is stuck, if the discussion gets out of hand, the inclusion of back-up questions, etc. The focus groups were conducted by one moderator, who was supported by two research assistants who made sure that the recording ran smoothly, and helped the moderator in making sure that no one was left out, etc. The focus group interviews had the following structure: First, participants were greeted and it was made sure that any questions or technical problems, for example, regarding the camera, sound, internet, were cleared. If everyone was ready to proceed, each focus group received a brief (4 slides) introduction to the concept of walkability by one of the helpers. This was done as it was a goal of the focus groups to discuss not only the concept of walkability, but to enable the participant to understand the concept, what it assesses, and what use cases can be derived. For example, participants were given information about what the variables (pedestrian network permeability, greenspace proportion, population density, and amenities available within walking distance) mean. After walkability was introduced, participants were asked a second time whether they were ready to start the focus group. If everyone was ready, participants were again asked for approval to start the recording, and the focus group commenced. The focus groups were divided into two main sections: While the first section had a focus on AM, the second section was used to collect information about SocPar. The two sessions were divided by a break of approximately 10 min.

## Data collection and data analysis

The focus groups lasted between 89 and 108 min (breaks not counted; G1: 1 h 29 min, G2: 1 h 48 min, G3:1 h 43 min) and were transcribed verbatim. Processing and editing of the transcripts and the data analysis were done using MAXQDA Plus [53]. Since there was a clear and structured approach to investigate and explore barriers and facilitators of AM and SocPar of different individuals from different urban neighborhoods, the analysis was based on categories that were created from thematic analysis (categories are predetermined). Open, axial, and selective coding was applied to the transcripts to ensure systematic analysis and interpretation of the data. This included the identification of patterns, issues, and relations between the different concepts and contexts. While investigating the transcripts, the memo function in MAXQDA was used to capture ideas and thoughts right in the manuscript to aid in the open, axial, and selective coding process. First, the transcribed data were compared and coded to categories that contained information regarding the research questions (open coding). Next, in an iterative process, possible connections, relations, and overlaps between the categories were investigated, to identify patterns or structures (axial coding). Last, the focus was once again on identifying and creating the main categories that contain the central aspects and key factors regarding the research questions (selective coding). Once all categories were created, definitions and concomitant exemplary quotes for each category were added to ensure transparency and reproducibility of the coding process [54]. The data analysis process was conducted by two researchers (LB, MK), who read and analyzed the interviews independently and discussed the findings (method of consent coding [55]). If necessary, a third researcher (CN) was included in this process for consultation, and to resolve any non-concordance. For better international understanding and consistency in terminology, all quotes that are important for this paper were translated from German to English using DeepL Pro (https://www.deepl.com) and then verified for accuracy by the authors. The translation of the quotations was carried out after the transcripts had been analyzed (July 2024) and the quotations had been selected for inclusion in the manuscript.

## Code categories

Multiple code categories were created to address the research objectives based on the focus group interviews’ transcripts. The first section focused on AM, and the second on SocPar. Based on social-ecological models, the categories were allocated to dimensions: One dimension consisted of factors and characteristics regarding the environment, with the categories (1) ‘Points-of-interest, infrastructure’, (2) ‘Safety, communication, community’, and (3) ‘Topography, physical compositions, weather, aesthetics’. A second dimension consisted of factors and characteristics regarding the individual, with the category (4) ‘Personal / individual attitudes, influences, evaluations’. This resulted in the categories depicted in Table 1. Note: A description of the content of categories 1–4 can be found in Additional file 2 for AM, and Additional file 3 for SocPar, respectively.

## Results

### Descriptive characteristics

A total of 17 individuals (7 women, 10 men) with an overall mean age of 43.4 ± 14,6 years (min-max: 21–64; 2 NA) participated in the focus groups. 11 individuals lived in objectively determined high walkability neighborhoods, and 6 in low walkability neighborhoods. For a detailed breakdown of the participants concerning age, sex, and residency in a high / low walkability neighborhood, see Additional file 4.

## Objective 1: Key factors of AM and SocPar

The collection of general key factors (barriers and facilitators) that residents stated to determine (non) engagement in AM and SocPar (research objective 1), resulted in the following findings. As is depicted in Table 1., the identified barriers and facilitators of both AM and SocPar could be assigned to the same dimensions: ‘Environment’, with concomitant categories (points-of-interest, infrastructure; safety, communication, community; topography, physical compositions, weather, aesthetics), as well as dimension ‘Individual’ and concomitant categories (personal / individual attitudes, influences, evaluations). Another finding was that many of the general key factors (barriers and facilitators) of AM and SocPar that were stated by the residents to influence (non) engagement in AM and SocPar were very similar and in many cases identical (e.g., availability of POIs, greenness, traffic). This means that AM and SocPar shared some key factors of (non) engagement. In addition, the identified factors were also very similar or identical to objective and subjective factors (barriers, facilitators) that research identified.

**Table 1:** Residents’ subjectively perceived key factors of AM^1^ and SocPar^2^ for different dimensions and categories

Dimension	Category	a) key factors AM	b) key factors SocPar^3^
Environment	1) Points-of-interest, infrastructure	Amenities (e.g., malls, markets, grocery stores, restaurants), greenspaces (e.g., parks, forests), public mixed-use areas, educational institutions, sidewalks, public transport, bike lanes (1, 2, 3)^3^	Amenities (e.g., malls, markets, grocery stores, restaurants), greenspaces (e.g., parks, forests), public mixed-use areas, sports- & playing fields, public pools, street-, and neighborhood festivities, public bathrooms (17, 18)
	2) Safety, communication, community	(Stationary) Traffic, traffic lights & duration, adequate lighting, separate lanes for each mobility form, coexistence and different speeds of different mobility forms (pedestrian, e-scooter, bikes, e-bikes, cars), sidewalk- & bike- & car lane width (4, 5, 6)	Traffic, -lights, -noise, cleanliness of public areas, neighborhood relationships and social cohesion, (pre) schools (for those with (in-) direct contact), availability & accessibility of information about events (19, 20, 21)
	3) Topography, physical compositions, weather, aesthetics	Weather (heat, sun, cold, rain, snow), design / layout, stationary traffic, slopes / hills, short cuts & discovery & exercise via Stäffele^4^, street aesthetics & characteristics (single / multi-lane roads, etc.), number of (lowered & flattened) sidewalks, bicycle lanes & stands, parking spots, development- & population density (7, 8, 9, 10)	Weather (heat, sun, cold, rain, snow), design / layout, stationary traffic, seating options, activities of any sort, structuring & atmosphere of public areas, distance to & amount- & speed of traffic from public areas, accessibility & proximity of locations (22, 23, 24)
Individual	4) Personal / individual attitudes, influences, evaluations	Preferences (e.g., physical exhaustion), (in) dependence on car / public transport, (in) convenience (e.g., duration getting from A to B), convictions (e.g., sustainability, health-promotion), stress & home-office (diversion, sitting compensation), trip duration, mindfulness of one another (11, 12, 13, 14, 15, 16)	Preferences (e.g., number of contacts, frequency of SocPar, private vs. public, one-on-one vs. groups), personal wealth, social interactions for well-being, characteristics (e.g., in- / extroverted), anonymity of the city, local idiosyncrasies, social structure (e.g., socioeconomic status) (25, 26, 27, 28, 29, 30)

^1^AM = active mobility; ^2^SocPar = social participation; ^3^Numbers at the end of the cells in brackets refer to the corresponding citation number, which can be found in Additional file 5; ^4^open-air stairs that connect streets of different altitudes, especially within neighborhoods

### Objective 2: High and low walkability neighborhood-specific factors of AM and SocPar

In addition to the generally perceived factors (facilitators and barriers) of AM and SocPar, high and low walkability neighborhood-specific barriers along with proposed improvements regarding AM and SocPar were investigated. In the following, first, the results for the dimension and corresponding categories concerning AM are presented, followed by the results for each dimension and corresponding categories concerning SocPar.

### AM: Dimension environment

#### 1) POIs, infrastructure

In sum, a certain number of POIs are available in both neighborhood types, but the accessibility and variety are greater in high walkability (1). Also, the basic infrastructure is given in both neighborhood types, but in high walkability, the infrastructure is often better developed, and multimodality (combining different options for mobility, e.g., first walk, then switch to a bike or train before walking again) is much more easily available (2: “*I am basically multimodal because we live in the city center*,* which means we use bikes*,* public transport*,* trains and we use car sharing. We don’t have a car*,* we have made a conscious decision against having one*.”). Important to mention is that in contrast to the objective benefits of high walkability neighborhoods, residents who live there state that AM can be inhibited, e.g., via noise- and air pollution and reduced quality of living from too high traffic, as well as too narrow traffic lanes and sidewalks (see also Table 2., which depicts a more detailed overview of similarities and differences concerning barriers and improvements for each dimension and category of AM). Note that the numbers (e.g., 1 and 2) in this and in the following paragraphs refer to the corresponding citations. The citations and additional information can be found in Table 3.

**Table 2:** Juxtaposition of high and low walkability neighborhood residents’ key barriers and proposed improvements for AM^1^

Dimension	Category	Subject	High walkability	Low walkability
Environment	1) Points-of-interest, infrastructure	**Barriers (similarities)**	High traffic; feeling unsafe in traffic participation (walking, bicycling)	
		***Barriers (differences)***	*Noise- & air pollution; narrow traffic lanes & sidewalks; too few and too narrow (separate) bike lanes / sidewalks*	*Bad access-*,* availability-*,* & variety of POIs*,* public transport*,* activities; long ways / distances; too few bicycle stands*
		**Improvements (similarities)**	Calm traffic; more & separate traffic lanes per mobility form	
		***Improvements (differences)***	*Further facilitate multimodal mobility & park and ride options; better communication of (traffic) rules*	*Increase availability and accessibility of public transport*,* POIs*,* (recreational) activities; more bicycle stands*
	2) Safety, communication, community	**Barriers (similarities)**	Missing- / inadequate infrastructure and resulting feeling of unsafety in traffic (too few & too narrow bike lanes & sidewalks; no built separation between- & conflicts between different mobility forms); disregard of rules by cars (regarding allowed speed, parking); parked cars result in restrictions of visibility at crossings	
		***Barriers (differences)***	*Much (stationary) traffic & complex traffic situations & concomitant limited visibility (especially at crossings); inadequate rule communication & lighting; number and size of crossings (danger); many parked cars; disregard of rules by e-scooter (speed*,* parking)*	*Missing mutual attentiveness in traffic; long waiting times at lights and crossings; too few traffic and parking controls*
		**Improvements (similarities)**	Facilitate mutual attentiveness in traffic; separate the different mobility forms; improve rule communication, e.g., via more & good visible traffic signs	
		***Improvements (differences)***	*Speed limit of 40 km/h; learn from other cities (e.g.*,* bicycle streets); ensure adequate lighting in all ways; holistic approach to traffic planning that considers all users equally*	*Reduce waiting time at lights & crossings (pedestrian); increase traffic control (speed*,* parking)*
	3) Topography, physical compositions, weather, aesthetics	**Barriers (similarities)**	Topography (basin with hills / slopes); much (stationary traffic); non-separated lanes for each mobility form (bikes, pedestrian); unflattened, unlowered, & too narrow sidewalks; (bad) weather; long waiting times at lights and crossings	
		***Barriers (differences)***	*High population- & building density with concomitant higher (stationary) traffic*,* inhibited traffic flow*,* & reduced attractiveness*	*Too few traffic and parking controls*
		**Improvements (similarities)**	E-bikes to overcome slopes/hills; reduce overall traffic; built separations of lanes for each mobility forms; lower-, flatten-, & widen sidewalks	
		***Improvements (differences)***	*Reduce traffic to improve air quality & attractiveness of public areas; learn from successfully implemented measures; improve public transport; create more seating options*	*Ensure adequate lighting in ways; increase vegetation / greening of public areas; improve rule communication*
Individual	4) Personal and individual attitudes, influences, evaluations	**Barriers (similarities)**	Physical exhaustion (e.g., topography, trip length); egoism of the different mobility forms (inattentiveness of the different traffic participants for each other)	
		***Barriers (differences)***	*Initiatives like bicycle roads are misunderstood / misused e.g.*,* by cars; storage / accessibility barriers*, e.g.,* bike in cellar; aversion of public transport; unawareness of possibilities for and convenience of AM; missing variety in POIs*,* amenities*,* public areas*	*Dependence on attractiveness / pleasantness of trip route*
		**Improvements (similarities)**	Increase knowledge about benefits of AM (e.g., health promotion, stress relief, convenience) and how to overcome barriers; facilitate mutual attentiveness in traffic	
		***Improvements (differences)***	*Ensure balance between nature and urbanity; facilitate multimodality; increase variety of POIs*,* amenities*,* public areas*	*Facilitate mind-shift concerning benefits of AM in the upbringing of individuals (early conditioning); increase pleasantness / attractiveness of trip route*

^1^AM = active mobility; Note: For better differentiation, similarities are depicted in regular font and *differences* in *italic* font

**Table 3:** Selected citations from the study participants concerning neighborhood-specific factors for AM^1^

AM	
Citation	Category, walkability
**1_G2T2_(00:01:51)**: “(…) good short distances, local amenities are also close by, good accessibility by public transport, but we have an enormous traffic burden here and that means an extremely poor quality of life due to the high noise levels we have as a result of the traffic and also the poor air quality. (T1 nods)”	1, high
**2_G2T2_(00:25:29)**: “I am basically multimodal because we live in the city center, which means we use bikes, public transport, trains and we use car sharing. We don’t have a car, we have made a conscious decision against having one.”	1 & 4, high
**3_G2T2_(00:25:29)**: “I have to say that I really like cycling, but it took me a long time to dare to cycle in the city center. I still think it’s extremely dangerous, there are simply no cycle paths, you’re constantly sharing the road with cars, you have to be extremely careful. And it’s also really dangerous to walk in the city center (…) where we live. That means that the [streets] really circle around us, so if we want to go shopping, we have to constantly cross lanes where a bunch of cars are driving, and for my child, she’s learning, of course it’s somehow positive, she’s learning to deal with traffic, but it’s an extreme strain that you constantly have (…).”	2, high
**4_G3T3_(00:16:11)**: “(…) it’s just too dangerous for me, too dusty, too dirty and too loud, and I would arrive at the office so worn out, so it’s actually not possible, and that’s actually the only reason why I decide to to take the subway, and it’s the traffic in particular that prevents me from taking some longer routes by bike, and I generally feel extremely unsafe when cycling in Stuttgart, because I always feel very hemmed in and often don’t feel safe.”	2 & 4, low
**5_G2T1_(00:28:27)**: “(…) the coexistence between cyclists, pedestrians and cars is often quite tense, that’s for sure (…).”	2, low
**6_G2T6_(00:36:42)**: “Some of the (…) traffic lights take a very long time to turn red, which is one thing, but they even do that on wider roads where two different traffic directions are separated and they are so fast that the changeover from green to red is so fast that you can hardly get from one side of the road to the other. (T2, T1 & T3 nod).”	3, high
**7_G2T6_(00:36:42)**: “I find it extremely annoying (…) for the promotion of a smooth flow of pedestrian traffic, if you now (…) have a 5-minute walk and then I have to add 10 minutes because I have three traffic lights in between, that’s not necessarily very effective. (T2 nods).”	3, high
**8_G1T2_(00:22:58)**: “(…) it’s really the population density, you can see that in Möhringen too. You can cycle much better there than down below, where the population density and the density of buildings is much higher.”	3, high
**9_G1T3_(00:18:57)**: “(…) I do it out of (…) conviction, because I just think it’s good and I don’t want a car. And I also deliberately walk up the stairs. So it’s also a form of fitness training (…) in everyday life. And I actually think it’s good if there are nice staircases, I don’t skip them.”	4, low
**10_G1T1_(00:14:21)**: “(…) when you go shopping and you know you have to walk up a hill again (…). Where you then think three times, am I going to do this (…) on foot or rather by car, when you know you have to do a lot of shopping and there is an uphill slope.”	4, low
**11_G2T5_(00:38:57)**: “[The] issue (…) of acceptance, (…) as Mr. T1 has already explained, is really a question of mentality (…), it’s always somehow a matter of conflict, and usually also a matter of conflict almost (…) from the stronger to the weaker, so in the end the motorist towards the cyclist and the cyclist towards the pedestrian. By the way, it’s interesting, I’ve often observed that when people change their mode of transportation, their mentality changes too, so the car driver who rails against the cyclist then rails against the car driver when he’s a cyclist (…). Without realizing that he’s playing different roles himself.”	4, high

^1^AM = active mobility; Note: The location of each citation in the corresponding transcript is indicated as follows: For example, 1_G2T2_(00:01:51) would translate to 1 = citation number 1; G2T2 = focus group 2, participant 2; (00:01:51) = time stamp in corresponding transcript. Some citations for this study have been slightly edited for improved readability

#### 2) Safety, communication, community

In sum, in both neighborhood types, there’s a high potential for conflicts between the different mobility forms (cars, bicycles, e-scooters, pedestrians) (3), some ways lack adequate lighting at night, and measures are needed to improve the (perceived) safety of cyclists and pedestrians, especially more bicycle lanes with a built separation to car lanes (4: “*(…) it’s just too dangerous for me*,* too dusty*,* too dirty and too loud*,* and I would arrive at the office so worn out*,* so it’s actually not possible*,* and that’s actually the only reason why I decide to to take the subway*,* and it’s the traffic in particular that prevents me from taking some longer routes by bike*,* and I generally feel extremely unsafe when cycling in Stuttgart*,* because I always feel very hemmed in and often don’t feel safe.*”). However, as high walkability neighborhoods often attract more individuals, this can lead to concomitantly more complex traffic situations and higher overall traffic that require more extensive measures to facilitate AM (5). Noteworthy is that residents from both high and low walkability propose to improve rule communication (e.g., more and good visible traffic signs). Also noteworthy, only residents from low walkability neighborhoods mentioned the solution to increase traffic controls.

#### 3) Topography, physical compositions, weather, aesthetics

In sum, the topography of the city (basin with concomitant hills and slopes), dependency on good / adequate weather, number of (stationary) traffic, availability of high-quality infrastructure for bicycles (bicycle lanes with built separation to cars and pedestrians, and bicycle stands) and pedestrians (lowered, flattened, and widened sidewalks, short waiting times at lights) are important in both high and low walkability neighborhoods (6; 7: ”*I find it extremely annoying (…) for the promotion of a smooth flow of pedestrian traffic*,* if you now (…) have a 5-minute walk and then I have to add 10 minutes because I have three traffic lights in between*,* that’s not necessarily very effective. (T2 nods)*.”). Also, while residents from both neighborhood types require general measures to improve conditions for AM, in comparison, high walkability neighborhoods’ objectively better conditions facilitate overcoming topographical barriers and car dependency (Table 2.). Notably, while the infrastructure for AM is often better in high walkability, high population- and building density were also mentioned to be a barrier for AM (bicycling) in high walkability. (8)

### AM: Dimension individual

#### 4) Personal / individual attitudes, influences, evaluations

Both low and high walkability residents pointed out that they engage in AM because of positive aspects such as stress reduction, health promotion, sustainability, and recreation (9: “*(…) I do it out of (…) conviction*,* because I just think it’s good and I don’t want a car. And I also deliberately walk up the stairs. So it’s also a form of fitness training (…) in everyday life. And I actually think it’s good if there are nice staircases*,* I don’t skip them*.”). Personal habits (e.g., routines like riding a bike to work) and personal preferences (e.g., physical exhaustion) are also important in low and high walkability. Furthermore, both groups share the wish for independence from car use (2), and some have an aversion to public transport. A particularity of high walkability neighborhoods is that residents from there also perceive their neighborhood as high walkability, and some deem AM the fastest and most convenient way to get from A to B. A particularity in low walkability neighborhoods is a seemingly higher willingness to engage in AM, and many residents self-select to live in and with the characteristics of low walkability neighborhoods, to have less traffic and more calm.

In addition, important context-specific findings were found. Concerning AM for leisure and recreation, differences in high and low walkability neighborhoods were that in high walkability, residents engaged more in AM for purposes of physical activity itself (e.g., jogging) with high usage of nearby green areas. In low walkability, residents engaged more in strolling and gardening activities, and to get from point A to B. Concerning differences in the context of errands and commutes, residents from high walkability neighborhoods have shorter ways to destinations of daily needs. Residents from low walkability neighborhoods more often use the bike, and have to consider topographical barriers when transporting things (10: “*(…) when you go shopping and you know you have to walk up a hill again (…). Where you then think three times*,* am I going to do this (…) on foot or rather by car*,* when you know you have to do a lot of shopping and there is an uphill slope*.”). In sum, both groups desire outdoor activities and closeness to nature and deem them as important factors to engage in AM. Notably, mutual attentiveness in traffic was considered to be of high importance for safe AM participation and in need of facilitation in both neighborhood types (11). Note: For a more detailed overview of similarities and differences concerning barriers and improvements for each dimension and category of AM, see Table 2.

### SocPar: Dimension environment

#### 1) POIs, infrastructure

In sum, (a few) public institutions (e.g., schools, churches) and green areas that facilitate SocPar are available in both high and low walkability neighborhoods. However, in high walkability neighborhoods, the preconditions and ease to engage in SocPar are much higher, due to the physical proximity and greater variety of public areas, places, amenities, and POIs. Still, both neighborhood types require measures to improve the (built-) environment, POI offerings, and the number of local festivities / events to facilitate SocPar (see also Table 4., which depicts a more detailed overview of similarities and differences concerning barriers and improvements for each dimension and category of SocPar). Notably, residents from both neighborhood types propose to increase SocPar offers specifically for older adults (12: “*And for older people*,* maybe also a (…) kind of meeting center*,* because I think that is*,* for example*,* a real problem for older people in our neighborhood*,* that they become lonely*,* that they (…) have no opportunity to meet other people who have the same interests if they are not organized in a church or some other way*.”). Note that the numbers in the brackets at the end of the results for each category refer to the corresponding citation number, which can be found in Table 5.

**Table 4:** Juxtaposition of high and low walkability neighborhood residents’ key barriers and proposed improvements for SocPar^1^

Dimension	Category	Subject	High walkability	Low walkability
Environment	1) Points-of-interest, infrastructure	**Barriers (similarities)**	Too few local events / street fests; too few possibilities for older adults except the church	
		***Barriers (differences)***	*Too few outdoor spaces for activities; not enough seating options; too little space for street fests vs. too much stationary traffic*	*Public / free lavatories; too few public areas*,* amenities / POIs that are also often too far away*
		**Improvements (similarities)**	Increase seating options & public / free to use areas to meet, gather, & for activities; facilitate local events; create more opportunities for older adults to engage in SocPar; reduce & calm traffic around areas where people meet & increase aesthetics (greenness, etc.)	
		***Improvements (differences)***	*Structural improvement of public areas (better dividing up*,* etc.); increase outdoor gastronomy in nice locations (nature*,* calm)*	*Implement free lavatories; more playing grounds*,* cafes*,* bakeries & other amenities / POIs*
	2) Safety, communication, community	**Barriers (similarities)**	Car traffic	
		***Barriers (differences)***	*Traffic at public areas inhibits SocPar (especially for families with playing children as increased attention is required); littering reduces sightliness of public areas; feeling unsafe due to inadequate lighting*	*Lack of or limited public space; misuse of neighborhood streets and ways as shortcuts by cars*
		**Improvements (similarities)**	Reduce car traffic	
		***Improvements (differences)***	*Reduce / ban cars from public areas; increase neighborhood social connections; create intergenerational places that allow for people with differing interests to gather; use social media to connect (like-minded) people*	*Ensure traffic rule communication and adherence*
	3) Topography, physical compositions, weather, aesthetics	**Barriers (similarities)**	Accessibility of (public) locations	
		***Barriers (differences)***	*Bad weather alternatives; basin-topography limits expansion and creation of public areas; inadequate shading; traffic close by; bad visibility of signs and information; surface sealing & concrete usage exaggerate heat island effect; unattractive public areas (no nature*,* mostly concrete)*	*Bad structure and layout of public places and squares that inhibit privacy*,* too few seating options*
		**Improvements (similarities)**	More public areas with varying seating options & offerings for activities	
		***Improvements (differences)***	*Increase shading; limit height of fences*,* hedges*,* & walls in neighborhoods; intergenerational & cross-interest events; ensure high quality & visually appealing public areas; reintroduce*,* make available*,* and create more access to natural constructs; calm traffic at / around public squares / places to increase attractiveness*	*Better selection of event locations for people to “stumble” upon them; improve structure and layout of public places and squares by better dividing them up to allow privacy instead of one huge*,* empty open area & to create varying utilization possibilities*
Individual	4) Personal and individual attitudes, influences, and evaluations	**Barriers (similarities)**	Intensity / amount of- & wanted variability in offerings for SocPar varies interindividually, some want / need more than others; anonymity of the city; keeping to themselves of high SES residents; social connection within the neighborhood ends after direct neighbors; local (Swabian) mentality of being introverted / keeping to themselves; difficult to join established groups	
		***Barriers (differences)***	*Selfishness of individuals; overabundance of options / too many offers*	*Some residents seem not interested in SocPar; preference for more calm neighborhoods vs. distance to & availability of POIs: pity one can’t have both; wealth: much can facilitate-*,* little can inhibit SocPar*
		**Improvements (similarities)**	Find ways to facilitate; educate residents about the importance of short social interactions, e.g., short, random, unplanned small talks; increase chances for strangers and those with differing interests to get into contact	
		***Improvements (differences)***	*Certain level of bustle at locations can facilitate gatherings and interest*	*While POIs etc. are in AM distance*,* embrace AM*

^1^SocPar = social participation; Note: For better differentiation, similarities are depicted in regular font and *differences* in *italic* font

**Table 5:** Selected citations from the study participants concerning neighborhood-specific factors for SocPar^1^

SocPar	
Citation	Category, walkability
**12_G1T6_(01:00:03)**: “And for older people, maybe also a (…) kind of meeting center, because I think that is, for example, a real problem for older people in our neighborhood, that they become lonely, that they (…) have no opportunity to meet other people who have the same interests if they are not organized in a church or some other way.”	1, low
**13_G2T4_(01:42:17)**: “Ok, for me social interactions are very important, I also enjoy chatting with my landlord (…) and my (…) flatmate in the stairwell for 5–10 min. I think it’s very important to run into people in the neighborhood and say hello.”	2, high
**14_G2T6_(01:28:44)**: “(…) the market square [is] a bit deserted and it’s very concrete-like, even if you buy an ice cream there, then maybe you walk somewhere else, and with the ice cream in your hand, there’s no real shade, no real green, I think that it’s maybe not necessarily a place that (...) really invites you to linger (…).”	3, high
**15_G2T1_(01:29:56)**: “(…) I know the market square in Vaihingen too, sometimes I get the feeling that places like that would benefit from being a bit more structured. The only square I know in Stuttgart that is really impressive in terms of size is Marienplatz. It works relatively well there, but for other things, it’s sometimes the case that if you had two or three corners where you weren’t standing in (…) the open space, it might be more pleasant for people to have a bench or something (…), it might be more pleasant for some people than a (…) huge square. (T2 nods)”	3, low
**16_G2T4_(01:39:01)**: “Yes, what Ms. T2 just said is also very true and important. On the subject of the heat island effect, cities are being built more and more with concrete, and topics such as more green spaces, more water in the city, we are in Stuttgart right on the Neckar, but there is no real access. (T2 nods) The Nesenbach is underground. We have a B10 that runs over the Nesenbach and it’s not allowed to drive on the right side because there is a canal underneath, because of heavy goods traffic. Of the topics, you actually have it here, also the access to various, yes, natural constructs, but somehow in a certain time, in the 60s, everything was flattened for the car-friendly city. (T2 nods).”	3, high
**17_G1T4_(01:17:46)**: “I would actually say that, in principle, I would always intuitively say that it is not so important to have a quick chat or contact with strangers, but I think that Corona has perhaps made us realize that it was more important than we thought. (…) that it’s actually nice to just have a quick chat with people you don’t know (…) I find these little encounters throughout the day more important than I would have expected and really nice too.”	4, high
**18_G2T1_(01:40:59)**: “Well, for the neighborhood where I live, where I really like to live because you are really close to the edge of the forest. But I have to say that I have given up hope there. But it would actually be very important to me to have offers, like in other parts of the city where the density of development is higher. Of course, that has disadvantages, but also the advantages that there are more people, so the choice of who you meet is greater, you have more shops or something like that, so that would be important to me, but in the end, I’ll stay where I am, but it’s sad that you can’t have both.”	4, low
**19_G1T6_(00:54:40)**: “You want to be left alone here. Everyone has high walls around them and no name on the bell, so you just want to be protected.”	4, low
**20_G1T5_(00:55:13)**: ”I think both positions are right somehow, I always feel like there are so many offers out there, but as a result I can’t see the forest for the trees, or the other way around, as the saying goes. So it’s incredibly difficult for me to identify the things that are interesting for me.“	4, high
**21_G2T2_(01:42:48)**: “I think it’s a bit different for us, compared to Mr. T1, because there are too many people, it’s very oppressive sometimes. It would be nice if it were more the neighborhood, if the people somehow moved closer together and you could see more clearly that “hey, we belong together in this neighborhood” (…).”	4, high,

^1^SocPar = social participation. Note: The location of each citation in the corresponding transcript is indicated as follows: For example, 1_G2T2_(00:01:51) would translate to 1 = citation number 1; G2T2 = focus group 2, participant 2; (00:01:51) = time stamp in corresponding transcript. Some citations for this study have been slightly edited for improved readability

### 2) Safety, communication, community

In sum, communication of and information about events and good connections within the neighborhood are relevant for most residents. Also, measures to improve communication, the quality of stay in public areas, and the facilitation of neighborly relationships and short social interactions are important to foster SocPar in both high and low walkability. However, in high walkability areas, greater possibilities and a higher variety of options make engagement in SocPar easier than in low walkability neighborhoods. Notably, residents from both neighborhood types consider a minimum level of familiarity and social interaction with direct neighbors as important for SocPar (13: “*Ok*,* for me social interactions are very important*,* I also enjoy chatting with my landlord (…) and my (…) flatmate in the stairwell for 5 or 10 minutes. I think it’s very important to run into people in the neighborhood and say hello*.”), and car traffic to be negative for SocPar.

### 3) Topography, physical compositions, weather, aesthetics

In sum, residents from both neighborhood types stated the importance of the design and composition of public areas for the quality of stay and SocPar (14: “*(…) the market square [is] a bit deserted and it’s very concrete-like*,* even if you buy an ice cream there*,* then maybe you walk somewhere else*,* and with the ice cream in your hand*,* there’s no real shade*,* no real green*,* I think that it’s maybe not necessarily a place that (...) really invites you to linger (…).*”). The availability of seating options, possibilities for activities, and the dependency on good weather to engage in SocPar in public areas were also mentioned. But, in general, high walkability public areas often have better designs and features (e.g., structure and division of seating options), compared with low walkability neighborhoods (15). In addition, while both types of neighborhoods enable a certain engagement in SocPar, high walkability offers a higher quality and quantity of public areas (e.g., more parks and squares that are well tended to). However, in both neighborhood types people see the need for measures to improve the overall design and features of public areas. Important to mention is also that high walkability residents mentioned that surface sealing, no access to natural constructs, and public areas that are fully made of concrete can inhibit SocPar engagement (16).

### SocPar: Dimension individual

#### 4) Personal / individual attitudes, influences, evaluations

In sum, personal attitudes, influences, and evaluations play an important role in SocPar and shaping social relationships. Additionally, there are individual differences regarding the preferences (e.g., meeting in public or private) and the amount and intensity of SocPar wanted in both groups (Table 4.). However, residents from both low and high walkability perceived short social interactions positively (17: “*I would actually say that*,* in principle*,* I would always intuitively say that it is not so important to have a quick chat or contact with strangers*,* but I think that Corona has perhaps made us realize that it was more important than we thought. (…) that it’s actually nice to just have a quick chat with people you don’t know (…) I find these little encounters throughout the day more important than I would have expected and really nice too*.”). Also, residents stated that fostering SocPar can lead to an increase in quality of living. Low walkability neighborhoods seem to be more attractive to residents who want the neighborhood to be calmer, and can also facilitate stronger social connections due to less population density and more familiarity in the neighborhood. However, car dependency to get to places to engage in SocPar, and few chances for random encounters can result in a feeling of isolation (18).

In addition, finding the right balance between SocPar and privacy is important. Also, it can be hard to join already established social groups (Table 4.). However, some low walkability neighborhoods have a (very) high neighborhood-level socioeconomic status (SES, determined via the average purchasing power of residents in a neighborhood; data purchased from microm (www.microm.de/daten/soziodemografie-oekonomie)), which is perceived by some as inhibiting SocPar voluntarily, as residents “self-isolate” because they are satisfied with what they have (19: “*You want to be left alone here. Everyone has high walls around them and no name on the bell*,* so you just want to be protected*.”). The stereotype that Swabian individuals (Swabia, a region in the southwest of Germany of which the study location is the capital) are introverted was mentioned in both groups. In addition, both groups stated the anonymity of the city to be both positive (being able to mind one’s business) and negative (hard to find new social contacts). Notably, contrary to the objective benefits of high walkability neighborhoods, it was stated that an overabundance of options can inhibit SocPar, as residents can have difficulties identifying what they are interested in (20: “*I think both positions are right somehow*,* I always feel like there are so many offers out there*,* but as a result I can’t see the forest for the trees*,* or the other way around*,* as the saying goes. So it’s incredibly difficult for me to identify the things that are interesting for me*.“). In line with this, in high walkability neighborhoods, too high population density can also inhibit SocPar (21). Note: For a more detailed overview of similarities and differences concerning barriers and improvements for each dimension and category of SocPar, see Table 4.

## Discussion

This study used qualitative data to investigate subjectively perceived factors (barriers, facilitators) of AM and SocPar in high and low walkability urban neighborhoods to better understand how residents perceive their neighborhood environment. In addition, neighborhood-specific barriers, improvements, and possible particularities for (non) engagement in AM and SocPar were explored to aid in the understanding of (non) concordance between objective characterizations and actual levels of AM and SocPar.

Due to the first finding, AM and SocPar have some similar influencing factors and possibly influence each other, meaning that AM can lead to an increase in SocPar, and vice-versa. This is in line with research that showed that higher levels of SocPar are associated with being less likely to be physically inactive [13], and increased AM leading to more social interactions [14, 15]. Also, the key factors of both AM and SocPar could be allocated to categories from the dimensions ‘Environment’ and ‘Individual’ of social-ecological models [21, 22]. Furthermore, where appropriate, this allowed the discussion of the results per category for AM and SocPar in combination, instead of separately, one after the other. In addition, and in line with multiple reviews (e.g [56–59]), residents identified POIs, infrastructure; safety, communication, community; topography, physical composition, weather, aesthetics; and personal / individual attitudes, influences, evaluations (see Table 1.) to influence (non) engagement in AM and SocPar. In principle, this match between the objective measures of the reviews and the factors identified by the residents indicates that the subjective perceptions of the participants of this study are in line with those identified in generally valid samples from high-income countries. Also, the identification of general factors pro / contra AM and SocPar in urban neighborhoods per se can be regarded as being feasible via the implementation of qualitative focus groups. In addition, several unexpected particularities like mismatch findings were found concerning the high and low walkability neighborhood-specific factors. Concerning such mismatches, much research calls for assessments that specifically focus on how individuals perceive and evaluate barriers and facilitators when interacting with the environment (e.g [23, 37]). In this context, research that combines various methods and data is suggested to be promising in delivering explanations for such inconsistencies [23, 49, 60, 61]. In this regard, the findings of this study revealed the following (research objective 2):

### Dimension ‘environment’

Concerning **AM**,** category ‘POIs**,** infrastructure’**, an important finding was that in line with systematic reviews ( [62], see also [63] for adolescents; and [64] for older adults) participants confirmed the objective advantages of high and disadvantages of low walkability neighborhoods: high walkability facilitates increased chances to engage in AM that is further facilitated by available multimodality options. Contrarily, low walkability is characterized by too few POIs, amenities, etc. and increased offerings and better access to public transport are required to facilitate AM. An interesting and striking finding was that residents from high walkability neighborhoods reported barriers like high traffic and narrow traffic lanes and sidewalks to inhibit AM. This is supported by Pucher and Buehler [65], who describe that, among other policies, traffic calming and safe and convenient infrastructure such as sidewalks and bike lanes are needed to encourage AM.

On the subject of **SocPar**,** category ‘POIs**,** infrastructure’**, findings were notably greatly similar to the barriers and improvements reported for AM: (Dis) advantages of high and low walkability were confirmed, and increased offerings and possibilities were proposed as improvements. Another important finding was that residents from both neighborhood types stressed the importance of increasing offerings and possibilities, especially for older people, to engage in SocPar. This is supported by the scoping study findings of Levasseur et al. [56], which stress the importance of considering the proximity to recreational facilities and resources in interventions that aim to foster SocPar and AM.

A walkability-independent result was found for the highly discussed and eminent topic **AM**,** category ‘Safety**,** communication**,** community’**: Both high and low walkability neighborhood residents reported being anxious to participate in traffic via AM, as they struggle with conflicts and dangers that result from the different mobility forms, for example, having to share the same lane (e.g., cars and bicycles). Residents from both neighborhood types repeatedly called for infrastructure improvements (e.g., separate lanes for each mobility form, especially for bicycles) and also proposed the solution to increase and improve rule communication. These improvements are supported by findings from Hackl et al. [66], which indicate that car-centered infrastructure and shared roads negatively influence bicycling and that the presence of adequate bicycle infrastructure encourages bicycling. Interestingly, while residents from both neighborhood types proposed to improve traffic rule communication, only residents from low walkability additionally stated the wish for more traffic controls, especially concerning parked- and speeding cars, highlighting the need for neighborhood-specific measures.

As to **SocPar**,** category ‘Safety**,** communication**,** community’**, an important finding was that residents from both high and low walkability considered a minimum level of familiarity and social interaction with residents from the same neighborhood to be important for SocPar. Considering the ongoing ‘epidemic of loneliness and isolation’ [67], focusing on neighborhood SocPar seems to be a very promising issue to allocate resources for improvements. Supporting findings from Small and Adler [68] argue that creating and increasing the availability and accessibility of public areas (e.g., POIs and amenities), especially in neighborhoods with few offerings, contribute to (unplanned) social interaction, the formation of social ties, and are beneficial in many more contexts.

Concerning **AM category ‘Topography**,** physical compositions**,** weather**,** aesthetics’**, an important finding was that residents from both high and low walkability neighborhoods consistently reported unflattened and unlowered sidewalks, shared lanes, etc. to be barriers to engage in AM. This is in line with another focus group study [69] that also identified bad sidewalk quality, having to cross large roads, poor traffic light coordination and concomitant long waiting times, and narrow sidewalks as barriers to AM (walking). Another striking finding was that residents from high walkability reported that too high population- and building density can also inhibit bicycling. Contrarily, Giles-Corti et al. [70] found residents from neighborhoods with a higher density to engage in more bicycling (and walking) compared with residents from a neighborhood with a lower density. This shows that objective factors like population and building density that are generally positively afflicted with walkability and AM can indeed be positive for walking. But, at the same time, they can be perceived as barriers and inhibit bicycling.

In respect of **SocPar** and the **category ‘Topography**,** physical composition**,** weather**,** aesthetics’**, ensuring good accessibility, quality of stay, and design (aesthetics, green, seating options, etc.) of public areas was highlighted by both high and low walkability residents as important to facilitate SocPar. A highly interesting finding was that residents from high walkability emphasized what were considered ‘past mistakes’ of city planners like making the access to natural constructs unavailable and sealing them off. These ‘mistakes’ were named in line with criticism and open questions about the reasons why most public areas are designed with “only concrete”, few seating options, and inadequate shading. These findings are supported by findings back from Whyte [71, 72], who laid out the positive influences of aspects like seating options, accessibility, and natural elements (trees, water) for visitability and livability of public areas, which in turn increase chances for (short) social interactions and SocPar. In addition, the positive influence of seating options for public areas has also been shown in an experiment, in which 23 men and 37 women rated manipulated color photographs of plazas that varied - among other characteristics - in the number of seating options available, and found seating options to improve restorativeness [73].

### Dimension ‘individual’

Findings for **AM**,** category ‘Personal / individual attitudes**,** influences**,** evaluations’** were the most complex, as perceived attitudes, preferences, evaluations, etc. vary greatly interindividually, greatly complicating their investigation. In line with the excellent review of reviews of Travert et al. [37], residents identified the important role that past experiences, for example, positive / negative, of engaging in a behavior, along with attitudes, motivation, self-efficacy, convictions, etc. play in how the environment is perceived. This could mean that an individual who has a long history of engaging in AM to get from point A to point B, and additionally considers it to be convenient, will more likely continue to engage in AM for such purposes, even if the individual’s neighborhood doesn’t promote AM. However, in the same context, this means that no matter how good the environment or the circumstances for AM are in general, if the individual isn’t “on board”, (s)he won’t engage in AM. Another very interesting finding was that residents from both high and low walkability neighborhoods emphasized their dissatisfaction with the lack of mutual attentiveness in traffic: For example, the shift of perspective and egoism in one’s mobility role, in which an individual in the role of a bicyclist is annoyed with pedestrians blocking “their lane”. However, when that same individual is in the role of a pedestrian, (s) he’s annoyed with bicyclists riding in “their way”, forgetting that it also takes the other role. Residents repeatedly mentioned this lack of mutual attentiveness to be of great importance, as concerning traffic safety, the key is everyone being aware and considerate at the same time, especially when engaging in AM.

In line with AM, for **SocPar**,** category ‘Personal / individual attitudes**,** influences**,** evaluations’**, the same difficulties in the investigation of personal / individual factors arose, because high intra-individual differences make it difficult to derive generally valid statements. However, an important result was that residents from both high and low walkability neighborhoods perceived short social interactions in everyday life positively. This is in line with research from Bollenbach et al. [74], who investigated person-place interactions of adults during everyday life walking and found positive associations of mental health with both (short) social interactions and greenness. This underlines the health benefits of SocPar in urban neighborhoods that are fostered when residents engage in AM. Another striking result was that some residents from low walkability identified neighborhoods with a high percentage of individuals with (very) high SES to ‘voluntarily self-isolate’ and keep to themselves. Residential self-selection often plays a very important role in understanding why individuals do (not) engage in a behavior or live where they live, provided they have the choice [75, 76]. For example, some high walkability residents may live in their neighborhood specifically because of the concomitant benefits (POIs, short ways, etc.) and characteristics (e.g., increased density and liveliness). This is in line with a study from Zhu et al. [77], who found that residents who deemed “ease of walking” as important and who moved to a walkable community had increased levels of physical activity, walking, social interactions, and neighborhood social cohesion. Contrarily, others may self-select to live in calmer neighborhoods with less traffic that are in turn often concomitant with fewer POIs, etc., and greater distances to them. Concerning the latter and SocPar, a study that investigated the moderating effect of walkability on the associations between AM, subjective neighborhood perceptions, and SocPar found residents from low walkability to have a higher reliance on AM to engage in SocPar: AM can be a way for low walkability residents to compensate the reduced availability of places to engage in SocPar [48]. Another major finding was that residents from high walkability stated that too many offerings, for example, amenities and POIs, can inhibit SocPar, as they can’t decide on what’s of interest to them. Concerning objective determinations of the suitability of a neighborhood for SocPar, this shows that ‘the more the merrier’ is not always true.

### Strengths and limitations

First of all, it’s important to note that the results have to be seen in the context of urban neighborhoods of high-income countries, because the preconditions and circumstances of AM and SocPar in urban environments are very different from those in more rural areas. Barriers and improvements differ in urban vs. rural neighborhoods, with usually greater possibilities to engage in AM or SocPar in urban neighborhoods [30, 78]. One strength is the study area: The distinctive basin-topography with concomitant variations in environmental characteristics (hills, slopes, stairs, etc.) and the inclusion of 11 different neighborhoods enabled the identification of many different facilitators, barriers, improvements, and particularities individuals experienced in different neighborhood environments, resulting in a large amount of information being integrated. Another methodological strength is the size of the three focus groups (*N* = 6, *N* = 6, and *N* = 5). They were in line with the median participant count (*N* = 5) of focus groups as identified in an analysis of 220 papers published in 117 journals [79]. This is considered the optimal size (*N* = 5 to 8) for non-commercial groups, according to a practical guide by Krueger and Casey [80]. However, this study is subject to the general limitations of qualitative research: While focus groups were specifically chosen to enable in-depth investigations of subjective and perceived factors of residents’ (non) engagement in AM / SocPar, this limits generalizability. Concerning the categorization of factors of AM and SocPar, it has to be noted that some of these factors can be assigned to more than one category in certain cases, but we have nevertheless decided to retain our categorization structure to ensure a consistent analysis. Another methodological aspect that has to be mentioned is that the focus groups were conducted online. While this may have entailed a little reduction in the interactive and discussional character of focus groups, it also facilitated scheduling and enabled the participants to participate from the safety / familiar surroundings of their homes. Also, the common problem of recruiting- / self-selection bias with only or mostly residents who are interested in the subject participating also has to be considered in the interpretation of the results of this study. A limitation of this study was that fewer participants from low than high walkability neighborhoods and fewer women than men participated. Also, it’s important to note that no older participants (65 years and older) participated in the present study, which should be taken into account when considering the results. Future studies should attempt to specifically include different population groups (older individuals, LGBTQI+, immigrants, varying SES), as they may provide valuable insights into group-specific factors of AM and SocPar, and because the findings could substantially differ between these groups: One example concerning older adults is participant G2T6 (age: 61), who mentioned short duration of green lights for pedestrians at crossings as a barrier for AM, (Table 3., 6_G2T6_(00:36:42)) and G2T1 (age: 63) nodded in agreement. It’s also important to note that it’s difficult to determine how neighborhood-specific residents’ statements were, as engaging in AM often equates to crossing multiple neighborhoods that can have different walkability, but individuals could make their statements based on the experience of the whole trip. This is in line with criticism of research that investigates the environmental and behavioral characteristics solely at the location where individuals live and not holistically, i.e., across neighborhoods and places where individuals engage in (health) behavior [81–83].

## Conclusion

Increased AM and SocPar have repeatedly confirmed health benefits, making it a promising and valuable health-promoting strategy (e.g., [67, 84]). The findings confirmed established key factors that influence individuals’ (non) engagement in AM and SocPar and revealed that many AM and SocPar factors are very similar, or even identical. The in-depth investigation of high and low walkability urban neighborhoods revealed additional valuable neighborhood-specific information. This information speaks against measures and interventions that aim to promote AM and SocPar by simply implementing a static ‘factors catalog’ of (objective) factors. Just because factors are identical does not mean they can be addressed similarly across different neighborhoods. While we found consensus on many factors, the specific approach to addressing these factors may still need to be tailored to the unique characteristics of each neighborhood. Instead, the inclusion of residents’ subjective perceptions is of great relevance because interindividual subjective perceptions can add greatly to the explanation of (non) concordance between objective environmental determinations and actual levels of AM and SocPar. It’s suggested that city planners and public health officials implement neighborhood-specific interventions that include residents’ subjective perceptions in the identification of needs for action. Furthermore, interventions aiming to promote AM and SocPar should be coordinated to tap synergies by addressing key factors that multiple health behaviors share – for example, AM and SocPar- and should include behavioral components, e.g., as provided by Traver et al. ([37]). For future research, it’s suggested to combine and implement different, (interdisciplinary) research methods to further our understanding of why and under which circumstances and conditions individuals do or don’t engage in health behaviors such as AM and SocPar. This is of great importance in the creation of (urban) neighborhood environments that promote the health of their residents.

## Electronic supplementary material

Below is the link to the electronic supplementary material.

Supplementary Material 1: Additional file 1_Interview Guideline (translated from German to English).

Supplementary Material 2: Additional file 2_Overview of the content of the categories with a focus on active mobility.docx.

Supplementary Material 3: Additional file 3_Overview of the content of the categories with a focus on social participation (SocPar).docx.

Supplementary Material 4: Additional file 4_Sex, age, and residency in a low or high walkability neighborhood for each participant.docx.

Supplementary Material 5: Additional file 5_Citations to validate general key factors for active mobility (AM) and social participation (SocPar).docx.

## Data Availability

The data generated and analyzed during the current study are published via the research data repository of the University of Konstanz, KonDATA: (DOI: 10.48606/mSpHIuBfHaeoznLS).

## Abbreviations

AM: Active mobility (moving from A to B using your own body, e.g., walking and biking for leisure, recreation, errands, transport, etc.)
SocPar: Social participation (being involved in activities that result in interaction with other individuals)
