# Supplementary material for: Qualitative exploration of determinants of active mobility and social participation in Urban neighborhoods: individual perceptions over objective factors?
Source: Arch Public Health. 2024 Oct 16;82:183. doi: 10.1186/s13690-024-01408-z (PMC11481444; doi:10.1186/s13690-024-01408-z)
Supplement: Supplementary file 1 — Supplementary Material 1: Additional file 1_Interview Guideline (translated from German to English). [file 13690_2024_1408_MOESM1_ESM.docx]

## Additional file 1. Interview guideline for the focus groups.

Note: The interview guideline was translated from German to English using DeepL (www.deepl.com).

| **Topic** | **Key questions, overarching questions**  *Mandatory questions*   - *- Indented questions are follow-up questions or in-depth questions* | **In-depth questions/alternative questions**  These questions are not asked initially and are only addressed if the topic has not been mentioned beforehand. |
| --- | --- | --- |
| **Part 1** | | |
| Introduction / Warm-Up  Rating + Walkability Ice-Breaker-Theme-specific | Please introduce yourself very briefly:  1) Please tell us what your name is, how old you are, and in which residential area/neighborhood you live.  And now that you have been introduced to Walkability by our colleague, please give your neighborhood a school grade from 1 very good to 6 + a short explanation |  |
| Collecting specific examples  Across groups | Stuttgart generally has a very high walkability or a high walkability value. However, the individual districts also differ greatly. High walkability does not always result in a high number of footpaths. How is it in your neighborhood?  1) To do this, let's collect the things you do on foot or by bike in your neighborhood and are there other things you could do?  2) Against the background of this collection, what are the arguments for and against walking or cycling these routes?  Now we are discussing what would actually be good and for whom...  3) What do you think would help or what would it take to get people in your neighborhood to walk/bike more?  We have now seen, and research shows, that it is not the same good/bad for everyone.  4) If you now try to look at this question from the perspective of different age groups, i.e. young people, families, single parents, adults and old people... What is good or bad for which age groups? Are there similarities or differences?  5) If you could change two things in your neighborhood in terms of pedestrian friendliness, what would they be and why?  Now it's again about whether pedestrian friendliness plays a role for you personally, i.e. is it important at all. And the question in this context is,  6) With regard to quality of life and well-being:How important is it to you that your neighborhood is pedestrian and bicycle friendly and that you take advantage of this? | Are there also personal things, e.g. likes, dislikes, or other reasons that you (do not) walk, cycle?What are they?  is it strenuous? because it is healthy? to get a bit of exercise?  Can you think of examples from other neighborhoods, past places of residence, that you think are particularly good?  not only what is missing, but also what is particularly good elsewhere?  Other opinions on this? How do you see it - the same or different?  Potential for conflict?  Security? |
| BREAK | | |
| **Part 2** | | |
| Collecting specific examples  Across groups  Rating | We would now like to talk about social participation and social interaction in the context of residential neighborhoods. What is important, what parameters, i.e. what are the possibilities or reasons, do social participation and social interaction play a role at all in terms of quality of life and well-being in the residential area? What conditions, including those in the surrounding area, could there be?  And the first thing we would like to do together with you is to ...  1) Here again, start by collecting:  What are such places?  What meeting places, e.g. playgrounds/sports grounds, squares, leisure facilities are there in your neighborhood that enable people to meet up?  2) Against the background of this collection, what are your personal arguments for or against using, visiting or not using it?  Now we need to discuss what would actually be good and for whom?  3) Building on the previous question, what do you think would help or what would be needed to improve opportunities for social participation in your neighborhood?  4) We know that not all offers and circumstances are the same for everyone, so ...  If you now try to look at this question from the perspective of different age groups or groups of people, i.e. young people, adults and old people... What is good or bad for which age groups? Are there similarities or differences?  Now it should also be about you personally and how important this is for you, namely...  5) With regard to quality of life and well-being:how important is the opportunity for social participation, for social interaction in your neighborhood and that you do/experience this?  6) Now that we have identified and discussed possible important aspects of “Sociability”: what grade would you personally give your neighborhood in terms of the opportunity for social participation/togetherness and why?  7) If you could change two things about the possibility of social interaction, what would they be and why, what would they achieve? | What are the factors that promote social interaction and what are the barriers?  Do you know general things or examples from other neighborhoods, or past places of residence, that you think are missing in your neighborhood?  Any other opinions on this? How do you see it - the same or different?  Conflict potential?  Security?  from 1 very good to 6 poor |
| **Conclusion and thanks** | | |
