# Supplementary material for: Qualitative exploration of determinants of active mobility and social participation in Urban neighborhoods: individual perceptions over objective factors?
Source: Arch Public Health. 2024 Oct 16;82:183. doi: 10.1186/s13690-024-01408-z (PMC11481444; doi:10.1186/s13690-024-01408-z)
Supplement: Supplementary file 2 — Supplementary Material 2: Additional file 2_Overview of the content of the categories with a focus on active mobility.docx. [file 13690_2024_1408_MOESM2_ESM.docx]

Additional file 2. Overview of the content of the categories with a focus on active mobility (AM).

| Dimension | Category name | Description |
| --- | --- | --- |
| Environment | 1) Points-of-interest (POIs), infrastructure | Consists of a pure collection of places to go, things to do, and availabilities that can be considered in the context of POIs (e.g., places, attractions, amenities, etc.) and infrastructure (e.g., public transport, sidewalks, bicycle lanes, etc.). |
|  | 2) Safety, communication, community | Consists of a pure collection of statements regarding (un) safety, (in) security, (in) confidence, (e.g., duration of traffic lights ), etc., as well as statements regarding conflicts, conflict potentials, and communication (of rules). |
|  | 3) Topography, physical compositions, weather, aesthetics | Consists of a pure collection of statements regarding characteristics of locations, surface conditions, etc. and the weather. |
| Individual | 4) Personal / individual attitudes, influences, evaluations | Consists of statements regarding self-interest, personal opinions, experiences, factors, reasons, etc. that the participants state to play a role in the decision pro and contra the engagement in AM. |
