# Supplementary material for: Qualitative exploration of determinants of active mobility and social participation in Urban neighborhoods: individual perceptions over objective factors?
Source: Arch Public Health. 2024 Oct 16;82:183. doi: 10.1186/s13690-024-01408-z (PMC11481444; doi:10.1186/s13690-024-01408-z)
Supplement: Supplementary file 3 — Supplementary Material 3: Additional file 3_Overview of the content of the categories with a focus on social participation (SocPar).docx. [file 13690_2024_1408_MOESM3_ESM.docx]

Additional file 3. Overview of the content of the categories with a focus on social participation (SocPar).

| Dimension | Category name | Description |
| --- | --- | --- |
| Environment | 1) Points-of-interest (POIs), infrastructure | Consists of a pure collection of places to go, things to do, and availabilities that can be considered in the context of social participation (e.g., places, attractions, amenities, etc.) or that can result in SocPar. |
|  | 2) Safety, Communication, community | Consists of a pure collection of statements regarding good/bad communication, conflict (potentials), (un) safety, (un) easiness, etc. in the context of SocPar. |
|  | 3) Physical compositions, aesthetics, weather, aesthetics | Consists of a pure collection of statements regarding characteristics of locations, aesthetic (un) pleasantness, place conditions, etc. and the weather. |
| Individual | 4) Personal / individual attitudes, influences, evaluations | Consists of statements regarding self-interest and personal opinions, experiences, factors, reasons, etc. that the participants state to play a role in the decision pro and contra the engagement in SocPar. |
