# Supplementary material for: Qualitative exploration of determinants of active mobility and social participation in Urban neighborhoods: individual perceptions over objective factors?
Source: Arch Public Health. 2024 Oct 16;82:183. doi: 10.1186/s13690-024-01408-z (PMC11481444; doi:10.1186/s13690-024-01408-z)
Supplement: Supplementary file 4 — Supplementary Material 4: Additional file 4_Sex, age, and residency in a low or high walkability neighborhood for each participant.docx. [file 13690_2024_1408_MOESM4_ESM.docx]

Additional file 4. Sex, age, and residency in a low or high walkability neighborhood for each participant.

| **Walkability low / high** | **Participant** | **Sex** | **Age** |
| --- | --- | --- | --- |
| low | G1T3  G1T6  G3T3  G3T4  G1T1  G2T1 | female  female  female  female  male  male | 51  56  51  28  NA  63 |
| high | G1T4  G2T2  G2T6  G1T2  G1T5  G2T3  G2T4  G2T5  G3T1  G3T2  G3T5 | female  female  female  male  male  male  male  male  male  male  male | 31  32  61  NA  45  38  26  53  21  64  31 |

Note that the participant labels translate as follows: For example, G2T2 = focus group 2, participant 2.
