# Supplementary material for: Qualitative exploration of determinants of active mobility and social participation in Urban neighborhoods: individual perceptions over objective factors?
Source: Arch Public Health. 2024 Oct 16;82:183. doi: 10.1186/s13690-024-01408-z (PMC11481444; doi:10.1186/s13690-024-01408-z)
Supplement: Supplementary file 5 — Supplementary Material 5: Additional file 5_Citations to validate general key factors for active mobility (AM) and social participation (SocPar).docx. [file 13690_2024_1408_MOESM5_ESM.docx]

Additional file 5. Citations to validate general key factors for active mobility (AM) and social participation (SocPar).

| Citation | Category, walkability |
| --- | --- |
| **Section AM** | |
| **1_G2T6(00:08:05)**: “We actually have a shortage of cycle paths, but everything else is available: buses, trams, the S-Bahn, shopping, the post office, the university, schools, whatever you could wish for, even restaurants, small ones with inner courtyards or something, it's all wonderful.” | 1, high |
| **2)_G2T1(00:47:39)**: “What I have noticed is that it helps in principle if you have a center where you can get the essentials. That means that if you are on foot, you should be able to do everything you need there, because if you think you have to drive somewhere to do something, you will get the rest somewhere else as well.” | 1, low |
| **3_G2T1(00:01:08):** “(...) the walkability there, I would describe as very bad, (...) because (...) there is not a single shop, the bus connection is every 30 minutes, that's a real disaster” | 1, low |
| **4_G2T3(00:02:47):** “The only thing I generally miss here in Stuttgart are cycle paths, cycle paths separated from footpaths. (G2T2 & G2T1 nod).” | 2, high |
| **5_G2T2(00:25:29)**: “I have to say that I really like cycling, but it took me a long time to dare to cycle in the city center. I still think it's extremely dangerous, there are simply no cycle paths, you're constantly sharing the road with cars, you have to be extremely careful. And it's also really dangerous to walk in the city center (...) where we live. That means that the [streets] really circle around us, so if we want to go shopping, we have to constantly cross lanes where a bunch of cars are driving, and for my child, she's learning, of course it's somehow positive, she's learning to deal with traffic, but it's an extreme strain that you constantly have (...)..” | 2, high |
| **6_G2T1(01:12:21):** “(...) the stationary car traffic has to be removed from the city. (T2 nods) Drive in, deliver, do something, car sharing, no problem. But the stationary traffic is depriving us of the open spaces we need to do it reasonably differently. (T5 & T2 agree)” | 3, low |
| **7_(G2T3.114)**: “But it's also a fact that (...) it's much easier to take part in traffic with cycle paths than without. (T6 nods) In Stuttgart, I somehow have the feeling that many cycle paths (...) have been crossed with pedestrian paths, there's just a sign saying that the path is shared by pedestrians and cyclists, and there's not even enough room for (...) this path, (T5 nods).” | 3, high |
| **8_G1T4_(00:29:09):** “(...) I just had this thing with the lowered sidewalk, I think that's something that would be good for all age groups, both for parents with strollers and for cyclists, (...) but then also (...) for older people with walkers [and] for people with walking disabilities in wheelchairs, all of that. (T3 nods) So you wonder why it's not at every traffic light, that's actually sad, and then also with the additional sidewalks, they're not just sidewalks, but often things on wheels are also on the move there, and yes, in that respect it would be a thing that runs through, I think (T2 and T3 nod).” | 3, high |
| **9_G1T4_(00:17:24):** “And the trivial point, the weather is of course also an issue, if it's really stormy and raining, you don't feel like going out, if you have your car parked outside, you might just take it. And the same applies now to extremely hot weather.” | 3, high |
| **10_G3T2_(00:02:09)**: “(...) the geological location here in Stuttgart is sometimes a bit bad to be able to ride a bike or walk.” | 3, high |
| **11_G2T4_(00:16:45)**: “Perhaps on the subject of why I do it, I find that it's simply the fastest way to get from A to B, to get things done. And of course there's also a bit of ideological background (...).”  **12_G2T4_(00:16:45)**: “The reasons for this are, on the one hand, because it is very easy for me to make the trips, and also the background that, for sustainability reasons, I prefer to choose the alternatives without costs, without CO2 emissions.” | 4, high |
| **13_G2T6_(00:36:42):** “I find it extremely annoying (...) for the promotion of a smooth flow of pedestrian traffic, if you now (...) have a 5-minute walk and then I have to add 10 minutes because I have three traffic lights in between, that's not necessarily very effective. (T2 nods).” | 4, high |
| **14_G2T5_(00:38:57):** “[The] issue (...) of acceptance, (...) as Mr. T1 has already explained, is really a question of mentality (...), it's always somehow a matter of conflict, and usually also a matter of conflict almost (...) from the stronger to the weaker, so in the end the motorist towards the cyclist and the cyclist towards the pedestrian. By the way, it's interesting, I've often observed that when people change their mode of transportation, their mentality changes too, so the car driver who rails against the cyclist then rails against the car driver when he's a cyclist (...). Without realizing that he's playing different roles himself.“ | 4, high |
| **15_G2T3_(01:02:44)**: “For me personally, it is very important that I have (...) good shopping facilities nearby, that I don't have too long distances, that I have good transport connections, (...) but also that my children are safe here (...) in the neighborhood, don't have to walk through dark alleys and [are not exposed to] traffic too (...) much.” | 4, high |
| **16_G1T3_(00:43:55)**: “So when it's green outside and I can walk and I can just stroll along at my own pace, so to speak, and see what's happening around me, that contributes a lot to my well-being and [then] I don't want to have to go just one stop, just out the front door and I have beautiful surroundings and it's green and I can just walk there, that's, I think, the highest relaxation effect right from the front door.” | 4, low |
| **Section SocPar** | |
| **17_G1T3_(00:32:40)**: “(...) [A] large square that is very green and there are these permanently installed table tennis tables, there are permanently installed tables, different benches, either on the square or under the trees, so in the green and it is very popular with children, young people, older people. So I think it's a good mix.” | 1, low |
| **18_G2T2_(01:20:41):** “Yes, we have a great meeting place in our neighborhood, (...) for young people there is a sports field, a football pitch, a skate park and also a playground for families with children, it's all so close together and it's not uncommon for people to get more in contact.“ | 1, high |
| **19_G1T4_(00:51:42):** “(...) often there is a lack of communication about where things are taking place, so I think you have to actively look for them.” | 2, high |
| **20_G1T4_(01:02:31)**: “Just one more thing about family-friendly things, (...) I also think that traffic plays a role here, if you have a place where there is no traffic, where you can just talk to your friends and let the children play there without having to worry. I could imagine that that is also important. (T6 nods)” | 2, high |
| **21_G1T5_(01:07:50):** ”Yes, but I mean, it's annoying when the (...) bin is right next to it (...) and people don't think it's necessary to throw the stuff in. But also when bins are there that aren't emptied for a long time and then the places where you are really become unsightly and you don't want to be there anymore.” | 2, high |
| **22_G1T2_(00:50:27)**: “But it's also important to look at what a neighborhood actually has to offer at different times of the year and in different weather conditions.” | 3, high |
| **23_G1T3_(01:02:55):** “Yes, as has already been mentioned, simply differently designed seating (T4 nods), so that you can make yourself comfortable, even sitting opposite or around the corner, and yes. Different accessibility (T2 nods) (...) maybe also like a small one, that you can climb up somewhere and sit there if you can physically, and the others just come to the bench with their walker (...) (T4 & T6 nod).” | 3, low |
| **24_G2T1_(01:29:56):** “(...) I know the market square in Vaihingen too, sometimes I get the feeling that places like that would benefit from being a bit more structured. The only square I know in Stuttgart that is really impressive in terms of size is Marienplatz. It works relatively well there, but for other things, it's sometimes the case that if you had two or three corners where you weren't standing in (...) the open space, it might be more pleasant for people to have a bench or something (...), it might be more pleasant for some people than a (...) huge square. (T2 nods)” | 3, low |
| **25_G3T2_(01:32:16)**: “(...) [the] wallet in this case would not necessarily promote social contacts, rather everyone is satisfied with what they already have, because they are already living well (...).” | 4, high |
| **26_G1T6_(00:53:19)**: “And I can't really say why, I just think it's the structure of the people. It's just not the kind of neighborhood where people move to meet and socialize.” | 4, low |
| **27_G3T2_(01:06:18)**: “Well, I think so, it's a bit also the Swabian mentality. So you warm up relatively slowly (...).“ | 4, high |
| **28_G3T5_(01:10:02)**: “I also have to agree with Mr. T2 that the Swabians take a long time to warm up to someone.” | 4, high |
| **29_G1T4_(01:17:46)**: “that it's actually nice to just have a quick chat with people you don't know (...) I find these little encounters throughout the day more important than I would have expected and really nice too.” | 4, high |
| **30_G2T4_(01:42:17)**: “Ok, for me social interactions are very important, I also enjoy chatting with my landlord (...) and my (...) flatmate in the stairwell for 5 or 10 minutes. I think it's very important to run into people in the neighborhood and say hello.” | 4, high |

Note: The location of each citation in the corresponding transcript is indicated as follows: For example, 1_G2T2_(00:01:51) translates to 1 = citation number 1; G2T2 = focus group 2, participant 2; (00:01:51) = time stamp in corresponding transcript. Some citations for this study have been slightly edited for improved readability.
